# Overweight and obesity: The serious challenge faced by Chinese children and adolescents

**DOI:** 10.7189/jogh.13.03036

**Published:** 2023-07-21

**Authors:** Zhenggang Zhu, Ping Yin

**Affiliations:** 1 School of Health Sciences, Universiti Sains Malaysia, Kubang Kerian, Kelantan, Malaysia; 2 Hunan Provincial Maternal and Child Health Care Hospital, Kaifu District, Changsha, Hunan, China

Overweight and obesity have become a serious public health problem in China, especially among children and adolescents. In 2015, China had the highest number of overweight and obese children globally [1], with almost 39 million obese children aged ≥7 years old [2]. This number is expected to reach 58 million by 2030 [3]. According to the 2021 Children’s Blue Book: China Children’s Development Report, the incidence of overweight and obesity among school-age children in China was 15.5% in 2010, rising to 24.2% in 2019 and soaring to 29.4% in 2022 [4]. The weight of children and adolescents in China seems to be increasing drastically, which is deeply concerning.

## THE HARMS OF OVERWEIGHT AND OBESITY ON CHILDREN AND ADOLESCENTS

Overweight and obesity significantly increase the risk of chronic diseases in children and adolescents. For example, while the incidence of hypertension in normal-weight children is between 3.85% and 4.10%, it is estimated to be 2.6 and eight times higher in overweight and obese children, respectively [5]. The prevalence of dyslipidaemia in Chinese children and adolescents is 25.3% [6], but the detection rate in obese children is as high as 50%, with a 2.6 times higher risk of elevated triglycerides than in normal-weight children [7]. Obesity can cause airway narrowing and airflow obstruction, and obese children are six times more likely to suffer from obstructive sleep apnoea syndrome than non-obesity children [8]. The prevalence of asthma in obese children is twice as high as in non-obese children [9]. Furthermore, obesity is the most important risk factor for type 2 diabetes in children and adolescents, likely due to the insulin resistance and β-cell dysfunction [10].

The global incidence of metabolic syndrome is 2.8% in overweight and obese children and 4.8% in adolescents [11]; in China, this incidence in these groups increases to 4.7% and 17.3%, respectively [12]. The prevalence of non-alcoholic fatty liver disease in Chinese children overall is 3.4%, but can reach 50%-80% in overweight and obese children [13]. Overweight and obesity also increase the risk of early puberty [14]. The excessive accumulation of fat in the body of obese children promotes the rise of oestrogen levels, stimulating the hypothalamic-pituitary-gonadal axis and accelerating the development of secondary sexual characteristics such as breast, leading to precocious puberty [14]. Obese girls have earlier menarche, and an increased risk of developing irregular menstruation and polycystic ovary syndrome [14]. Childhood and adolescent obesity also increase the risk of various skeletal and muscular diseases, including limited mobility, increased risk of fractures, lower limb joint pain, and poor limb alignment [15].

**Figure:**
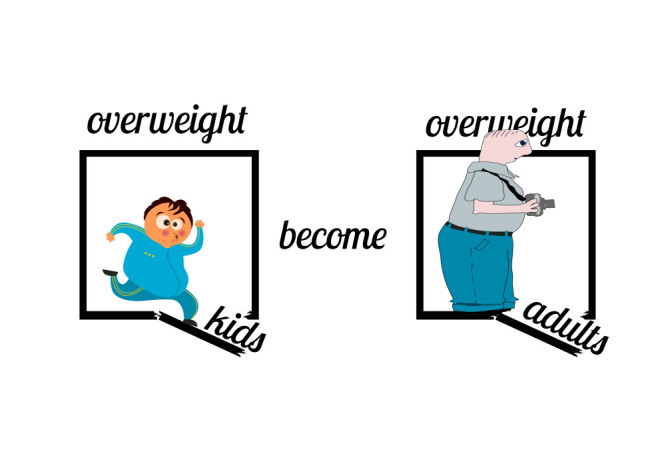
Photo: Obese children are more likely to remain obese later in adult life. Source: Gerd Altmann, Pixabay. Available: https://pixabay.com/illustrations/remove-overweight-children-adults-1050369/. Free to use under Pixabay license.

**Figure 1:**
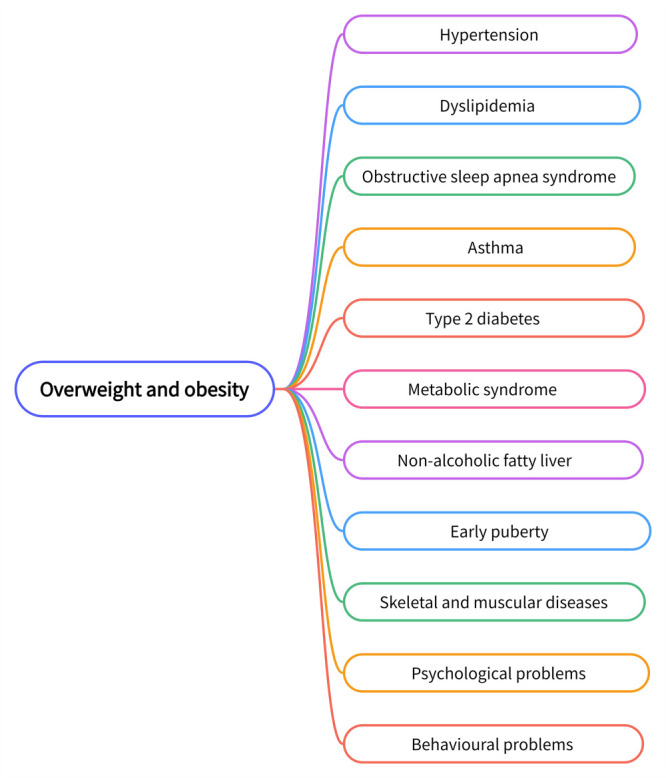
Overweight and obesity increases chronic diseases in children and adolescents.

Besides potential physical health problems, psychological and behavioural ones are also prominent in obese children [16]. For example, due to obesity, they may be excluded from group activities by their peers as well as be stigmatised and ridiculed [16]. These acts of discrimination damage their self-esteem and make them less confident. Over time, individuals may develop unhealthy personalities, exhibiting traits such as aversion, emotional irritability, impaired self-awareness, low self-esteem, non-conformity, unhappiness, dissatisfaction, and poor social adjustment (**Figure 1**).

## WHY ARE THERE MORE AND MORE OVERWEIGHT AND OBESE CHILDREN ANS ADOLESCENTS IN CHINA

The increasing number of overweight and obese children and adolescents in China is inextricably linked to the economy, society, and culture. With China's rapid urbanisation and large-scale population migration, the proportion of urban population in China increased from 19.4% in 1980 to 65.2% in 2022 [17]. Urban expansion has reshaped the work and lifestyle of ordinary people. In rural areas, labour-intensive jobs such as farming, logging, and animal husbandry have been replaced by low-physical-exertion jobs in the manufacturing and service sectors. In 2022, the proportion of overweight and obese adults in China reached 50.7% [18].

Genetic inheritance is an important risk factor for childhood overweight and obesity [18]. The probability of a child becoming obese is two to three times higher when one parent is obese and 15 times when both parents are obese [19]. The proportion of overweight and obese Chinese adults continues to increase, significantly increasing the probability of obesity in the young generation. Additionally, Babies born with excessive birth weight are also a risk factor for overweight and obesity in children and adolescents [20]. Therefore, it is recommended that pregnant women should not over-supplement nutrition during pregnancy.

Changes in the dietary structure also contribute to the problem. With the rapid development of the Chinese economy and improved living standards, people’s dietary habits have shifted towards more animal-based foods, refined grains, and highly processed foods [21]. With accelerating lifestyles, more families are eating out at restaurants instead of cooking at home. Restaurants often serve high-fat, high-sugar, and high-calorie dishes to attract customers. Eating unhealthy food outside is a common cause of obesity.

Moreover, in 2022, 200 million children were born from one-child families in China, with one-child families accounting for 40% of all families [22]. The parents overindulge the only child, who is given more pocket money to buy snacks and carbonated beverages, leading to excessive nutrient intake [23]. Additionally, with the rapid development of the internet and express delivery companies in China, ordering food online has become more convenient. More and more families and adolescents are choosing to order food online – usually unhealthy junk food delivered within the hour – which further contributes to childhood overweight and obesity.

Physical inactivity and increased screen time are also major problems today [21]. According to the 2017 Physical Activity and Fitness in China report, approximately two-thirds of the total 131 859 students aged 7-19 years failed to comply with the World Health Organization's recommendation of engaging in at least 60 minutes of moderate-to-vigorous physical activity per day, while a third did not meet the guideline of limiting their screen time to less than two hours per day [24]. With a high academic burden from excess homework, children and adolescents significantly reduce their time for physical activity [25]; they also increasingly own smartphones and tablets and use electronic devices for socialising, playing video games, and attending online classes, significantly increasing sedentary screen time [24]. Consequently, they have less and less time to participate in extracurricular activities. Moreover, excessive academic pressure has become common as students strive to perform well in exams, leading to stress-induced obesity [25].

## OBESITY-RELATED NATIONAL POLICIES AND PROGRAMMES

The Chinese government and health authorities have implemented several measures to address this epidemic of overweight and obesity [3]:

1. Actions to promote physical activity (such as Happy 10 Minutes, Sunshine Sports Activities, and Walking action for professional people);
2. Nutrition and school-based actions (such as China Student Nutrition Day, National Nutrition Campus Program, Nutrition and Health Action for Early Life 1000 Days, Student Nutrition Improvement Action, Student Health Education Action, and Healthy Children Action Plan (2018-2020))
3. Comprehensive actions (such as the National Healthy Lifestyle Action Plan (2017-2025) and the so-called Three Reductions and Three Health Action (i.e., reduction of salt, sugar, and oil, and promotion of healthy weight, healthy oral health, and healthy bones)).

Recently, China has introduced new policies to promote public health, some of which are not explicitly targeted at preventing and controlling obesity, but rather include obesity-related strategic and operational objectives. In October 2020, China announced a strategic plan for controlling and preventing obesity in children and adolescents [26], aiming to reduce the annual growth rate of childhood overweight and obesity during 2020-2030 by 70% compared to 2002-2017. The plan also requires primary and secondars schools to ensure that their students engage in at least three hours of high-intensity physical activity per week and emphasises the importance of healthy diets and physical activity. It also highlights the responsibilities of parents, schools, medical institutions, and the government in overcoming this epidemic [26]. In 2021, the State Council issued the China Children Development Program (2021-2030) [27], and in 2022, the Expert Consensus on the Diagnostic Assessment and Management of Childhood Obesity in China (2022) was published [28]. These programmatic documents provide clear guidelines for preventing and treating childhood obesity.

## HOW SCHOOLS AND PARENTS CAN ACT TO OVERCOME OVERWEIGHT AND OBESITY

Overcoming overweight and obesity in children and adolescents requires action from schools and parents, including monitoring and early prevention, as obese children are more likely to remain obese later in adult life. Despite the Chinese government's efforts to address the obesity epidemic, rates of overweight and obesity among children and adolescents continue to increase. We believe that schools and parents need be more attentive of the government's policies to make them more effective.

Schools play a crucial role in shaping students' behaviours and habits. To combat overweight and obesity, schools should change the way they evaluate students' excellence, focusing less on test scores and more on physical fitness. They should strictly follow national physical education and health curriculum standards and include students' physical fitness in their evaluation criteria, as well as provide students with sufficient physical activity time, such as at least two hours of outdoor activity per day for kindergarten children and one hour of medium-to-high-intensity physical activity per day for middle and primary school students. Nutrition and health classes should also be integrated into the curriculum to increase students’ knowledge of balanced diets and physical activity.

To improve the dietary structure in schools, we suggest that schools improve the quality of the food supply, develop school meal guidelines, hire professional chefs, and provide food that meets the nutritional needs of children and adolescents. This includes offering fresh vegetables, fruits, coarse grains, fish, poultry, meat, eggs, and dairy products, while avoiding high-sugar, high-fat, and high-salt foods.

Parents and caregivers should also take responsibility for their children's health, be attentive of their children’s nutritional intake, ensure food diversity, and reduce frying and deep-frying while controlling oil, salt, and sugar intake. Parents should encourage physical activity and create a positive family sports atmosphere by participating in sports activities with their children, guiding them to outdoor activities and physical exercise and encouraging the development of regular physical exercise habits. Reducing sedentary time watching electronic screens and ensuring sufficient sleep time is also important. Through joint action, schools and parents can help combat overweight and obesity in children and promote healthy lifestyles.

## CONCLUSION

Overweight and obesity among children and adolescents are becoming a serious public health challenge in China, which, if not effectively controlled, will seriously harm the health and growth of minors and bring serious burdens to society. The United Nations Sustainable Development Goal (SDG) 2 explicitly calls for attention to children's nutritional status, elimination of hunger, and reduction of obesity. The COVID-19 pandemic from 2020 to 2022 has resulted in China implementing strict home quarantine and other restrictive measures, which have reduced outdoor activities among adolescents, making the problem of obesity more prominent [29]. Fortunately, the pandemic is over, and there are still seven years until the realisation of the SDGs goal in 2030. Governments, schools, families, communities, and relevant departments must join forces and actively participate in the campaign against overweight and obesity.
